# Expertise, Identity, and Relationships in Private Forestry Practice

**DOI:** 10.1007/s11842-022-09537-5

**Published:** 2023-03-07

**Authors:** Abigail L. Jamison, Theodore R. Alter, Allyson B. Muth

**Affiliations:** 1 Forest Resources Planning Section, Pennsylvania Dept. of Conservation and Natural Resources Bureau of Forestry, Harrisburg, PA USA; 2 Department of Agricultural Economics, Sociology, and Education, The Pennsylvania State University, University Park, PA USA; 3 Department of Ecosystem Science and Management, The Pennsylvania State University, University Park, PA USA

**Keywords:** Consulting Foresters, Forestry Practitioners, Interpersonal Connections, Local and Expert Knowledge, Private Forest Landowners

## Abstract

In the management of private forests, it is important for a forester to understand and prescribe action rooted in sound science and technique but also in landowner values. Prior research has indicated that storytelling and sharing, which surface a landowner’s values, often result from an established, trusted connection with their forester. Empirical and experiential knowledge indicate that landowners and foresters who engage over prospective forest management often fail to create such a connection, leaving landowners feeling less trusting of the recommendations coming to them and vulnerable to the outcomes of potential unsustainable practices proposed by other actors. Such disconnect could be related to conflict between how landowners desire to engage foresters and the ways traditional forestry practice has prepared foresters. To further understand the landowner-forester disconnect and possibilities for overcoming it, this paper analyzes landowners’ interactional experiences with consulting foresters. Interviews with landowners indicated that professional and personal identities, as well as landowners’ perceptions of foresters, impacted the formation of a productive, working relationship between them. In their stories, landowners described that finding personal connections had the power to change their perceptions and catalyze the relationship. Results underscore the importance of allowing expert and personal identity to surface in order to create a relationship, informing the way in which foresters carry their expertise. This research suggests that being a forester is about being more than an expert, but also about being a knowledgeable and empathetic collaborator.

## Introduction

“Now we have biologists, environmental scientists and others trying to do forestry, calling themselves foresters…but the forester used to be king…” – Seasoned consulting forester reflecting on what he sees as one of the largest failures in the occupation at a professional meeting.

“…One of the guys… just acted as if I had no idea what I was talking about, and just blew me off when I would ask him a question…That’s just not the right way to go about things if you’re trying to establish a relationship with somebody.” – A landowner describing prior experiences with consulting foresters as part of this research.

The quotes above represent two different perspectives of forestry practice. Yet both convey conflict about two central questions: Who holds forestry expertise? And who is involved in forestry practice? These quotes suggest that landowners and foresters may answer these questions differently: foresters, by training and practice, are equipped to be the expert in successful forest management, while landowners are confident in their lived experience on their land and have insight regarding how it should be managed.

Perceptions of self and roles in forestry expertise play out in the nature and success of landowner-forester interactions with consequences for forest management and health. Some interactions are short-term and transactional – to fill a pragmatic or economic need. Other landowners desire to create a longer-term stewardship relationship with someone who will help guide the improvement of their forestland for years (Knoot et al. 2009; Jamison and Muth 2022). While central to the latter interactions, strong relationships between landowners and foresters can result in positive, sustained interactions even when the need is transactional. Yet, prior research has demonstrated that these interactions can fail in establishing strong relationships (Gootee et al. 2010; Jamison and Muth 2022). This is problematic in that the absence of “thick,” deeply interpersonal engagement between foresters and landowners potentially creates openings for practices inimical to private forestland sustainability. This leads to the following question: what if forestry practice was not only about solely being the scientific, technical expert, but also about utilizing mutual, respectful, and interpersonal engagement – within which forestry expertise can be shared and received – as a vehicle for successful management and the mutually-productive long-term relationships vital to it?

The purpose of this paper is to challenge the nature of expertise and practice within the forestry community and to analyze landowner-forester interactions and relationships in the context of Pennsylvania private forestland ownership, informed by landowners’ experiences and perspectives. Interactions and relationships were investigated empirically. Perceptions of oneself and others are at the heart of this investigation, particularly that the alignment of said perceptions affects relationship development (Elliot et al. 2003). We posit that formation of respectful, collaborative landowner-forester relationships is impacted by the perceptions these parties have of themselves and each other and the ability to reframe those perceptions by finding personal commonalities.

This work is set in a gap of the existing literature. We see the benefits of good relationships between landowners and foresters, leading to improved best management practices (Egan 1999; VanBrakle et al. 2013; Wilson et al. 2022) and increased income potential and quality of forest products harvested (Bullard and Moulton 1988; Hubbard and Abt 1989; Chhetri et al. 2018), as framed in “expert-client” roles – the forester has technical expertise that they share with a landowner to guide management practices (Knoot et al. 2009, McBride et al. 2019). This research creates an understanding of the relationship from the landowner perspective – what they want, what works for them, and encourages them to practice good forestry on their land, a space we do not see clearly elucidated in existing literature.

We begin by sharing stories of private forestland ownership in Pennsylvania and traditional forestry practice, reflecting on the conflictual expectations within them. We then discuss the nature of perceptions and how personal identity, alongside perception of others, can overcome or enable relational disconnects. Following methods, findings suggest that the ability to navigate away from expert identity is important to establish relationships with landowners and that, more broadly, a reflective, relational forestry practice model can be more effective than the traditional model. We conclude with reflexive observation regarding the nature of practitioners’ expertise and responsibility to it. Despite its challenge to conventional wisdom and culture, our work is devoted to strengthening the efficacy, equity, and longevity of forestry practice, and does *not* disrespect the essential, critically important expertise required for scientifically sound forest management. We are evaluating the question of how we *carry* that expertise into our interactions and throughout our practice. A broader reflection upon this question is not isolated to forestry; our intent is to provoke us all to reflect upon how we approach our work. As you read, we ask that you welcome any personal tension that arises and use it as a tool to evaluate what you, personally, take from this work and how it relates to the way that you carry your expertise and identity into your daily practice. This paper uses quotes and stories derived from the interviews conducted to illustrate themes, as well as to provide background on Pennsylvania private forestland ownership. The broader research inquiry on which this article is based focused on exploring the nature of landowners’ experiences with their advisors, both peer and professional (Jamison and Muth 2022). Here, we focus solely on landowner relationships with consulting foresters.

## Background

### Pennsylvania Private Forestland Ownership and Traditional Forestry Practice

Almost seventeen million of Pennsylvania’s twenty-eight million acres are forested (USDA Forest Service 2020). The last iteration of a state-wide survey of Pennsylvania landowners indicated that for parcels one acre in size and larger, the state has over 738,000 landowners, owning 70% of the total forestland (Metcalf et al. 2012). Within the contiguous United States, approximately 58% of forestland – 443 million acres – is privately owned. Pennsylvania ranks third in number of forest landowners, and these comprise 5% of landowners nationwide (Butler et al. 2016). Pennsylvania forest landowners get to know their land as they experience it. Pennsylvania-based research has demonstrated that they develop an understanding of their forest, alongside a deep connection to it, identifying themselves and their land as part of one another (Metcalf et al. 2012). A desire to care well for the forest – a stewardship ethic (Knoot et al. 2009) – arises out of that connection (Egan and Jones 1993; Erickson et al. 2002; Jamison and Muth 2022) but can be tempered by a lack of understanding of how to manage that resource. Caring for the forest can include practices like improving wildlife habitat, controlling non-native vegetation, and more (Butler et al. 2016; Metcalf et al. 2012). In seeking to implement more intensive management, landowners can turn to professional foresters. When trying to engage someone who has proper expertise, most landowners, whether out of relational or transactional need, hope to hire a professional who understands and shares their intrinsic value and land connection (Erickson et al. 2002; Gootee et al. 2010; Jamison and Muth 2022) or who can help them accomplish specific goals (Andrejczyk et al. 2016; Chhetri et al. 2018).

For example, drawing from this research, Cassie and her family own land that has been in the family for decades. They want their forest to be healthy and resilient. When invasive plants threatened, they sought a professional. For Cassie, the role of her consulting forester, Eric, was to serve as a “sounding board” for the family’s ideas, and to willingly educate them so they could play an active role in managing their forest. Cassie also placed high value on working with someone who would consider the importance that she ascribes to the land. She has spent “…two to three weeks [there each year her] entire life and it is [her] favorite place in the world.” This place is part of her narrative as a person, and she wanted her forester, Eric, to understand that and embrace what it meant for caring for the land – and her – well.

Also from this research, after purchasing his grandfather’s property, Bryce and his wife Krysten got to work updating structures and utilities. Bryce’s attention shifted to the forest when a tree fell on his truck. He looked for a professional who could remove hazardous stems, help the forest be healthy, and do it the right way – where a balance of delicate care would also allow for monetary returns. After interactions with some foresters and loggers, he was hesitant about hiring either – he felt they were dishonest, lacking forest management knowledge and empathy for his goals, and would be willing to bend the legal “rules” of forest operations. When Bryce met Curtis, a consulting forester, and Curtis’s knowledge, passion, and willingness to work for Bryce’s goals became clear, he was pleased to hire him. Bryce also values honesty and respect, communicating from that value with openness and candor. Bryce was grateful when he saw that Curtis communicated in the same manner and read in him a similar personality. They were “kindred spirits,” as he said, and Curtis even reminded Bryce of his grandfather. He took comfort in this and trusted Curtis because he could see that Curtis cared for the property.

Cassie and Bryce have different stories and objectives. Cassie’s needs are relational, and Bryce’s more transactional. It is the deeply personal nature of owning, connecting to, and caring for land that binds theirs, and others’, stories together as a collective tale of forestland ownership. Whether seeking a long-term professional partner or someone to remove timber for now, most landowners want to work with a forester who can and does go beyond a technical perspective of forest management – who will engage in their unique situation with an interpersonal understanding of their values and a willingness to welcome their knowledge as imperative for management action (Knoot et al. 2009; Gootee et al. 2010; Kittredge et al. 2013; Fischer et al. 2019; Jamison and Muth 2022). Moreover, they seek to create a relationship with their forester that is based on shared personal understanding – in that there is comfort and trust that the forester is working in care of the land with the owner as a collective endeavour (Jamison and Muth 2022).

Traditional forestry practice, however, is storied by the forester as expert more than as partner or collaborator. Most of the profession’s 130-year tenure has seen foresters trained in programs that prioritize technical (e.g., silviculture, dendrology, mensuration) and general education (e.g., speaking and writing, social science, humanities) competencies for entry level success (Redelsheimer et al. 2015; Sample et al. 2015). A call for people-oriented education, going beyond generalities, surfaced in the late 1960s, yet by the 1990s, studies indicated that forestry graduates continued to lack competencies like communication, leadership, collaboration, and conflict resolution (Brown and Lassoie 1998; Sample et al. 1999). Even in the last decade, curriculum has remained deficient in these topics (Meyer 2020) and employers have expressed that recent forestry graduates are unprepared for interpersonal and communicational tasks, and the socio-political environment inherent in their work (Sample et al. 2015). Training has prepared professionals to serve as technical experts but has less successfully integrated general and social competencies necessary to succeed as partners. This is reflected in the language some foresters use today to describe their perceptions of landowners.

Prior to this research, members of our forestry community have reflected on the difficulty of being anything but the expert: at a workshop to improve interpersonal skills, one forester said, “But I can’t take off that hat. I can’t not be the expert” (Muth pers. comm.) In participant recruitment, a consulting forester declined to provide a client for interviewing, stating, “There’s not enough control on my part…I think the forester should be able to sit in [on landowner interviews] and help them get the story right because we are like an attorney. Would the attorney want the suspect to not answer questions properly?” (Jamison pers. comm.). In sharing this research’s findings, many foresters reflected on the following sentiment: landowners often don’t know what they want or care about when managing their forests. These examples illustrate a perception of the forester’s authority without granting autonomy or credibility to landowners; yet landowners seek professionals and collaborators who are open to and respect their knowledge, understand their values, and are willing to develop a relationship with them (Gootee et al. 2010; Fischer et al. 2019; Jamison and Muth 2022).

Placing the stories of forest landowners and traditional forestry practice side-by-side, it is evident that what the human dimensions of forest management require and what traditional forestry practice brings are in conflict. To see this conflict more fully, we must understand how these perceptions, and the ability to change them, can influence landowner-forester interactions. For this, we draw on concepts elucidated in framing theory.

### Perceptions Impact Relationships and Trust in Expertise

As described in framing theory, we interpret the world, a situation, ourselves, and others by comparing current experiences with past ones (Gray 2003). As we encounter someone or something new, we create a perception or characterization about that thing/person based on prior experiences (Gray 2003). Relationships are a function of our perceptions of “the other” and ourselves (Gray 2003; Palmer 2011). When our perception of who someone is – their identity – matches our perception of ourselves – our identity – we are more likely to engage, trust, and form a deeper connection (Elliot et al. 2003). We discover shared identity via personal conversation – storytelling, asking questions, and speaking to life beyond expertise (Schön and Rein 1994; Elliot et al. 2003). If our initial perception or characterization of someone’s identity conflicts with our sense of self, and conversation has not facilitated understanding and/or better alignment of identities, we are less likely to engage further with that person. Others, depending upon their perceptions of us as individuals, may experience a similar dynamic (Schön and Rein 1994; Fischer 2000; Putnam and Wondolleck 2003; Palmer 2011). In light of misaligned identities and perceptions of “my” characterization and “your” identity, and vice versa, relationships fail to deepen, trust is unlikely to establish, and two parties are unlikely to believe each other’s narratives.

Previous research (Lewicki et al. 2003; Gootee et al. 2010; Kittredge et al. 2013) has shown that breakdowns in communication and failed landowner-forester relationships occur when perceptions that one has of the other conflict with one’s own or the other’s identity. Because perceptions are gatekeepers for relationships – and relationships are crucial to landowners’ trust in expertise vis-à-vis the application of forest management (Gootee et al. 2010, Jamison and Muth 2022) – it is imperative for foresters and landowners to shift their perceptions of each other from expert/landowner to a shared identity where understanding and connection are likely to be found. These shifts and discoveries are embedded in and emerge from the interactions between people; therefore, we posit that perceptions present within landowner-forester interactions impact the nature of said interactions and the efficacy of relationship formation. The ability to reframe perceptions as more than “professionals” or “clients” may serve to mitigate landowner-forester disconnects. The ultimate goal of the interaction is effective forest management and care – relationships are critical input into that process.

The following analysis focuses on interactions between landowners and professional foresters and was guided by the questions: What characteristics of interactions led to the formation of effective, sustainable relationships and what does this mean for professional practice?

## Methodology and Methods

### Phenomenology as a Methodology

Phenomenology asserts that lived experiences shape perceptions and realities (Thomas and Pollio 2002). Essentially, people are social beings, and interactions with the world – people, situations, things – inform a person’s understanding of reality or truth, and, consequently, their behavior. What is meaningful or true to a person about their experience is reflected in the story they tell of it (Pollio et al. 1997). To understand the true nature of landowner-forester interactions – and what was meaningful to the construction of relationships – we needed to uncover the experiences of those who lived them. This methodology exemplifies engaging in a reciprocal, collaborative, democratic model vs. a traditional, expert-based model of research practice. By asking landowners to detail their experiences and honoring their agency to share their stories, while listening with empathy, we gain insight into their values, beliefs, and perceived reality (Flyvbjerg 2001) regarding their interactions with foresters.

### Methods

To capture these stories, we conducted phenomenological and semi-structured interviews with forest landowners. Phenomenological interviews, which utilized unstructured and descriptive conversation, uncovered the important elements of an interactional experience between a landowner and forester via the landowner’s story. The researcher opened the conversation with a single question, and all follow up questions were derived from the story shared. Semi-structured interviews followed with thirteen prepared questions and uncovered demographic information as well as additional support in uncovering key points about the landowners’ backgrounds and relationships with consulting foresters. Lastly, field notes were recorded post-interview. These served to reflect upon the interview itself, but also on researcher positionality. In preparation for the interviews, we made explicit our assumptions about what we would hear by bracketing (Pollio et al. 1997), a process through which we surfaced, and continued to surface via field notes, our assumptions to ensure they were not coloring what landowners’ stories were telling us.

### Sample Selection

Potential participants were novice forest landowners (NLOs henceforth): people who own forestland and have no formal training in forest management. NLOs had recently interacted with a consulting forester^1^.

Participants were recruited by the consulting foresters with whom they interacted. Consulting foresters on the Pennsylvania Department of Conservation and Natural Resources Consulting Forester Directory were contacted and asked to share a written invitation from the Finley Center for Private Forests at Penn State with NLOs fitting the above criteria with whom they had interacted. It is important to note that in Pennsylvania, there are no forest practices laws or regulatory certification systems that utilize a professional standard to delineate foresters from the greater field of practitioners. However, inclusion in the aforementioned directory requires a forester to have received a degree in natural resources management. This ensured that NLOs who participated had worked with an individual qualified, via their education, as a forester or forest manager.

NLOs who wished to participate contacted the researchers directly or asked the forester to share their contact information.

Seventeen Pennsylvania NLOs participated. Per Penn State COVID-19 and IRB Human Subjects protocols (IRB# STUDY00014878), interviews were conducted virtually and were recorded upon participant approval.

### Analysis

Interview types were analyzed separately and through different mediums. Phenomenological interviews were analyzed by an individual researcher and research working group. The group, comprised of qualitative researchers, acted as a source of validity (Thomas and Pollio 2002) – they collectively analyzed transcripts and discussed findings across interviews. The individual researcher performed cross-checks by comparing group findings to those of independent analysis as well as to the broader findings of related meaning across all other interviews. Broader findings gave rise to themes that indicate ways in which stories of interaction bore experiential similarity to each other (Pollio et al. 1997) and represent broad level understanding of what was most meaningful to landowners within the interaction. The themes represent aspects of the experience for all participating NLOs. Validity was achieved by sharing results with participants, who confirmed them as representative of their lived experiences. Semi-structured interviews were analyzed independently, in a similar iterative manner, using Nvivo computer software. Results provided additional support for the phenomenological findings.

### Potential Limitations

While the strength of this research using a phenomenological methodology allows us greater insights into relationships, we recognize potential limitations in our methods.

Due to the nature and timing of this research, we were only able to capture one side of the interaction – that of the landowner – to understand their perceptions. Future work is planned to elucidate the other side of the interaction – that of the professional.

We also recognize concern over the small sample size, which may have implications for generalizability. However, in phenomenology, appropriate numbers of study participants are determined by the number of interviews completed before themes begin to emerge. Usually, six to twelve interviews are appropriate (Thomas and Pollio 2002; Pollio et al. 1997; Polkinghorne 1989).

Our method of identifying NLOs was limited in its capacity to capture stories of challenging or negative experiences. To recruit participants, we relied upon the group of advisors to provide landowners with whom they interacted. Recruiters were inherently more likely to invite landowners with whom they perceived they had had a positive experience. However, these NLOs’ stories highlighted aspects that are desired by landowners, which becomes a means of informing the presentation of expertise and engagement by the profession.

## Results

NLO participants were diverse in their experiences, knowledge, and backgrounds. Ownerships ranged from six to 4,000 acres, but despite their lack of formal training, NLOs’ confidence in their forestry knowledge was consistent. Regarding race, ethnicity, age, and gender, this sample was less diverse: NLOs were predominantly male, white, and between 40 and 90 years of age. However, this sample is representative of the larger forest landowner population of Pennsylvania (Metcalf et al. 2012).

Five themes emerged from interviews: *Trust*, *Forester as Partner*, *Developing Interpersonal Landowner-Forester Relationships*, *Forester Attitudes and Actions*, and *Skepticism as Context for Landowner-Forester Relationships*. These themes from across our sample are illustrated using representative quotes drawn directly from the participants’ experiences with consulting foresters. Identifying information has been changed and pseudonyms used.

### Trust

“We were kindred spirits…We agreed on a lot of things, and just by reading his actions and what he did, I felt that I could trust him, and I felt that his interests were in the right spot…I felt that he had the knowledge and the passion, and I trusted him.” – Bryce.

Trust was imperative to a NLO-consulting forester interaction. NLOs perceived risk in inviting someone onto their land for management, particularly because they did not have the technical knowledge to assess the work.

This mattered because NLOs knew that, if not cared for well, they and their land would face the consequences. Therefore, being able to trust foresters to leverage their knowledge towards land care, and not what NLOs perceived as ulterior motives (i.e., professional monetary gain at the expense of the forest), was integral to a NLO’s willingness to believe a forester’s expertise. Being able to believe their forester provided comfort to enter a relationship with them. This relationship began with the forester fulfilling many roles – including that of a partner and advocate.

### Forester as Partner

NLOs saw their foresters filling various roles. They recognized their foresters as educators, explaining the rationale of forest management decisions. While NLOs regarded foresters as experts, with the training and knowledge to manage forests, they also saw their foresters as partners.

In this partnership, NLOs welcomed their foresters’ knowledge and guidance as a learning tool and appreciated when foresters were mutually welcoming of their knowledge. Out of this, NLOs expressed their sense of agency over management projects stemming from their foresters’ respect and willingness to involve them as such. Frank describes this, saying,

He understood very early in the discussion that I was going to be involved in the process…I was going to put a lot of time [into participating], at least until I was certain that he understood what my goals were and…if I had conflicting goals…he would put enough time into explaining things to me from a professional aspect…to come to a path that worked for both of us.

Regardless, the clear trustworthiness and like-mindedness of their foresters assured NLOs that they would also play the role of advocate, upholding their values and mitigating their concerns throughout the process. When that like-mindedness also included similar identity beyond forest management, NLOs and foresters established valuable interpersonal relationships.

### Developing Interpersonal Landowner-Forester Relationships

For many, partnership advanced to interpersonal relationship when NLOs identified favorable character in their foresters and were able to discover personal similarities beyond the scope of their roles as foresters and landowners.

Character traits such as honesty, integrity, respect, and humility were valuable to NLOs, and they needed to uncover them within initial contacts to develop trust integral to connection. They conveyed this by relating it to the value they place on these traits in their lives. When asked about what it meant to have trust in his forester, Dennis said,

I probably draw on this experience because of being a contractor…, the number one thing people pay for with me is that they can trust me and…my honesty…I would like to tell you that I am the world’s best contractor…[but] you’ll hear that story from 15 other contractors…I think foresters are similar. They will give you their spiel…but you have to read what the forester *feels* (emphasis his) about the land.

Regarding other similarities, NLOs valued seeing similar personality traits (e.g., forthrightness, responsiveness, passion, patience) and similar experiences or interests (e.g., parent, outdoorsperson) in their foresters, indicating a shared identity. Similarities were uncovered as foresters and/or NLOs shared personal stories.

These relationships were vital to NLOs placing confidence in their foresters – because their actions would align with the NLOs’ values and perspectives, especially as they relate to or are borne out of similar identities. Because of these, Frank described that he and his forester Craig spoke the same language:

I knew Craig as a parent of a soccer player. I’m the parent of a soccer player. So…even though our kids were four or five years apart in the sport, I saw that. I saw a level of support there that seemed consistent with the way I was trying to support my child…

Creating a relationship and working and learning together led NLOs to feel they accomplished their goals and were satisfied with their foresters; but to arrive here, NLOs first had to assess the ability to trust and connect with them via observing their foresters’ behavior.

### Forester Attitudes and Actions

NLOs paid particular attention to their foresters’ behavior. It mattered to NLOs that their foresters were responsive to communication, interested and empathetic toward NLOs’ values, and that their foresters could communicate that they would prioritize NLOs’ goals. It also mattered that their foresters patiently listened to NLOs, were considerate in how they communicated their complex science, and did not act too busy for explanation. Such behavior left NLOs feeling that their foresters would uphold their values, be willing and able to work relationally towards said goals, and meet expectations for a satisfactory engagement. NLOs who had unsatisfactory experiences with their foresters cited misaligned expectations of each other’s roles, purpose, and character – which negated relationship formation and the overcoming of skepticisms – as the source for their dissatisfaction.

### Skepticism as Context for Landowner-Forester Relationships

It was notable for NLOs how their current interactions differed from a context of skepticism within which they had placed their expectations of how the interactions would go. NLOs consistently reflected on their experience relative to prior perceptions of others whom they believed to be forestry professionals. Recall that there are no legal or regulatory certification systems to a professional forestry standard in Pennsylvania; anyone practicing in the forest can legally call themselves a forester, logger, and so on. We cannot verify that the forestry professionals with whom NLOs had prior interactions are trained foresters who would meet such a standard of practice. This lack of professional accountability and distinction creates confusion among landowners about who is a forester, further amplifying a desire to deeper understand and trust their consultants and potentially contributing to their perceptions of these individuals. These perceptions involved skepticism relating to honesty, motives, and practice of loggers, foresters, and the broader forestry community. As related by Ron:

But there are some…[that] just want to get the lumber…to the mill and collect their check and that’s it…They don’t care what happens to your property once they’re gone…some of them I think [are] plain, downright dishonest in terms of calculating values and…the offers they make. And I think a lot of times they take advantage of people.

This skepticism was founded on past primary and secondary experiences. Primary experiences entailed varied interactions with others who they perceived as or who presented themselves as forestry professionals. Many NLOs talked about receiving communications from multiple loggers or foresters which presented divergent, and, at times, nonsensical, monetary offers for their timber. Some shared about discussions with foresters and loggers who regarded management in primarily economic terms, and not in terms of the NLO’s goals. Others spoke of foresters and loggers suggesting unlawful operations, like using equipment in wetlands or during poor weather conditions. To quote Dennis, these direct experiences,

…painted the picture that most of the people in the timber business are crooks…[this] caused me to be very wary…[and] very apprehensive about even thinking about cutting, because you almost know that you’re opening up a vulnerability to being ripped off or being taken advantage of.

Secondary experiences involved indirect exposures to the result of unfavorable interactions, such as stories from neighbors or friends or seeing examples of outcomes on other properties and feeling they “didn’t want that to happen on my land.” The consensus among NLOs of secondary experience was that, if not careful, a person could be easily taken advantage of by a professional.

Prior experiences, skepticism, and apprehension further amplify NLOs’ desire to quickly establish trust and connection with their foresters. When that connection was made and foresters hired, satisfaction in the foresters’ work was overwhelming when contrasted against the skeptical context.

## Discussion

Our broader inquiry revealed that interpersonal relationships are essential to conducting collaborative long-term forest management (Jamison and Muth 2022). This paper has focused on factors central to creating effective, sustainable landowner-forester relationships, and asserts that the discovery of shared values, beliefs, and interests – similar identities – dictates the likelihood that trust and consequent relationships establish at all (Schön and Rein 1994; Elliot et al. 2003; Gray 2003; Palmer 2011). In this section, we detail identities of NLOs and consulting foresters reflected in our sample and discuss how NLOs’ initial perceptions of foresters negated communication and trust, inhibiting relationship formation. Then, we discuss the process used to overcome negative perceptions and find common identity. We reflect on the implications for consulting foresters’ practice and conclude with the important responsibility that foresters have to their own and others’ knowledge and expertise within their own practice.

### The Forester, the Landowner: Different Identities from the NLO’s Perspective

When NLOs approach an interaction with consulting foresters, they identify as a landowner, who holds a deep connection, intimate understanding, and value for their land, alongside a responsibility to protect it (Metcalf et al. 2012). Will shared that he and his hunting camp members “…have a lifestyle [of] people living to take care of the forest… It makes [us] feel more a part of it, rather than it’s a piece of property. And the appreciation for what’s there is amazing.” As outlined previously, foresters approach the interaction identifying as the expert, wherein they perceive themselves equipped with the sound science, skills, and highly specialized expertise that enables them to guide forest management (Knoot et al. 2009, Andrejczyk et al. 2016; Chhetri et al. 2018; McBride et al. 2019; Wilson et al. 2022). Both parties also enter the interaction with perceptions of each other.

NLOs’ perceptions of consultants are revealed here and in other work (Gootee et al. 2010; Kittredge et al. 2013) and are shaped by what landowners interpreted as both hierarchical and potentially dishonest behavior. Regarding the hierarchical stance, many NLOs had prior experiences with foresters whom they perceived as domineering in the presentation of expertise, describing them with words like arrogant, disinterested, and inconsiderate. Al said

…They’re somewhat disinterested, because they just feel as though they have all of the right answers. And possibly because it’s not a field…that [I] work in that, instantly [they think] …as the landowner, I’m naive to things … where that’s not the case. Obviously, I’ve been in the outdoors my whole life…I know trees.

Perceived this way, NLOs feel that foresters grant little to no respect or value to the experiential knowledge that they identify as having.

The context of skepticism created a heightened need for NLOs to change their perceptions before trusting foresters. Ron, who had previously interacted with a forester, offered his perception of this need:

You have to make…decisions before you hire a forester. It’s in those first couple of meetings, and so I think these are things you need to look for: the character of the man…and the honesty…You have to determine whether you are that [same] type of man…So I would say, [try] to find out a little bit about the forester.

When the foresters in these experiences presented only their expert identity, and nothing to indicate a more personal identity, it negated NLOs’ abilities to discover a source of trust or connection in the person so that they might disprove their skepticisms about foresters (Schön and Rein 1994; Elliot et al. 2003). Even as foresters might have been seeking to do good management on behalf of their clients, and work antithetically to the skepticisms, NLOs’ perceptions of them were unchanged without evidence to suggest otherwise.

### “Kindred Spirits:” Reframing with Common Identities

NLOs and foresters conquered their identity mismatch by reframing their perceptions. Through storytelling and sharing about each other’s lives or interests, NLOs and foresters were able to discover the presence of similar identities, such as those created by being a parent, an outdoorsperson, a religious person, and more. When this occurred, the “expert professional forester” and “private landowner” identities were momentarily put in reserve, and these other more personal identities came to the forefront (Schön and Rein 1994; Elliot et al. 2003). NLOs and foresters were then able to understand one another as more than just foresters or landowners, reframing their perceptions of each other accordingly. Again, this research details the new perceptions NLOs created; while foresters likely also developed new perceptions of NLOs, this work cannot speak to that portion of the reframing dynamic.

Jeff, raised on and still operating a dairy farm, demonstrated this reframed perception of his forester, Henry:

I think…we connected [when] he was telling me...he gets up early in the morning and feeds his farm…he has a small farm…So he saw the setting where we were and he understood…it was like, ‘Yeah, okay.’ (laughs) I understood what he was going through and, and he knew I had a knowledge of that.

Being able to see that Henry had another identity as a farmer allowed Jeff to feel that Henry understood his knowledge of trees and land, as well as his values of prompt action, forthright communication, and hard work that stem from his own experiences as a farmer. From then on, Jeff analyzed Henry’s actions and suggestions regarding forest management through his new perception of Henry as a hardworking, honest man “like me.” Jeff was also one of the few participants who had a purely utilitarian and transactional need from his forester; clearing forest to erect a structure. Even still, it mattered to Jeff that his engagement with Henry was relational.

Importantly, when NLOs identified with their foresters, they were able to confirm that the foresters do not fit within the skeptical, hierarchical perception that they hold for others in the field. As Will said, it was a “night and day” difference between his forester and his perception of other foresters.

Through new perceptions that created shared understanding, NLOs felt ready to trust their foresters’ guidance and develop mutual, interpersonal relationships. Al believes, “When you meet and get to talk to somebody with the same interests that you have…there’s common ground where you can have a basis for a relationship.” Annie, when asked to elaborate on her sense of trust in her forester, quickly and firmly replied, “If you don’t have trust, you don’t have a relationship.”

With this trust, NLOs were willing to allow their foresters’ expertise to guide their understanding and collaborative work – even if that expertise challenged a NLO’s prior thinking or goals. Once Ron “…developed a trust in [his forester Eric’s] character and judgment, [he] felt comfortable with any advice Eric gave [him].” Bryce, when asked what it meant to him that his forester Curtis had similar personality traits and ethics, said, “It feels like you’d be able to have a little faith in what he [says], and [that he’s] willing to work with you.” NLOs also understood moments when their foresters offered a challenge, redirection, or suggestion as a learning opportunity coming from a trusted educator and advocate who sought to benefit the NLOs and their land.

Situated in their newfound connection, foresters and NLOs were free to move between their shared personal identities and roles as foresters and landowners/clients because there was an assurance that everything said or done matched the standards or values ascribed by that shared identity (Elliot et al. 2003).

### Implications: What Does this Say About a Forester’s Practice?

These findings suggest that a consulting forester is likely to be more successful in securing jobs with NLOs, or any landowner, if they are willing to present more of themselves than just the piece that defines them as the expert, to pinpoint personal similarities and share about them in initial conversations. This reveals the forester’s humanity to landowners and is likely to elicit the relationship wherein the forester has more freedom to share their expertise from their position as a forester and a person who is “like me” in the eyes of landowners.

While many landowner-forester interactions are one-time, transactional events, this research shows that landowners still desire deeper connections to the people guiding these activities. The perception of that person as trustworthy will likely result in professional recommendations to landowner peers, and ensure that the forester is contacted again, when the next forest management decision point occurs. From a business model perspective, creating interactions that lead to potential continued engagement, even five- or ten-years down the road, presents a level of sustainability for the practice itself.

This research also has implications for how foresters, as experts, carry their expertise. While landowners are seeking professional advice and guidance when they hire a forester to help them manage their land, the presentation of that expertise as all-knowing or one-sided becomes off-putting to the landowners. This research, and what came before (Gootee et al. 2010; Kittredge et al. 2013), indicates that landowners prefer to be invited into a forester’s expertise – to have it presented as a shared learning experience, with questions willingly answered, landowner lived experience considered, and solutions reached that incorporate scientific and technical expertise as well as landowner values and knowledge. This framing is consistent with the concept of democratic professionalism – wherein professionals share autonomy with and seek to understand the experience of their clients and utilize their own expertise alongside that of their clients to achieve a common goal (Dzur 2018). This concept could potentially serve as a successful alternative to the traditional expert model of forestry practice.

### Future Research

These findings highlight the need for future research to create a more complete understanding of the relational and interpersonal dynamics between landowners and foresters. To achieve this, it will be imperative for future work to uncover consulting foresters’ interactional and relational perspectives. Additionally, landowners often interact with various forest managers, depending on their needs. Therefore, future work could also focus on how identity and perceptions impact landowner interaction with other forest managers (i.e., service foresters, procurement foresters, Extension foresters, loggers).

Lastly, this research did not consider externalities, like opportunity costs of time associated with building sustainable interpersonal relationships and a consultant’s need to be employed on jobs that achieve necessary economic returns to maintain their business. This research has potential to inform foresters’ fee structure decisions regarding fairly and appropriately incorporating time for relationship-building within their paid practice.

### Responsibilities in Practice

This work does not suggest that foresters should turn their back on their expert knowledge of forest management. Rather, it suggests that foresters have responsibility to the land *and* the people they serve to present their scientific, technical knowledge as well as knowledge of themselves authentically, professionally, and personally.

Foresters care for the land with an intimate knowledge of how it works and functions ecologically – they have a specialized understanding that not many others do. When landowners engage with their land for many years, they get to know it and how it behaves across conditions. Yet landowners hire a professional because they recognize that they do not have the specialized scientific knowledge necessary to conduct big projects. They see gaps in their knowledge and seek an expert, hoping that the expert also recognizes their own gaps – particularly that they potentially cannot immediately know the landowner’s land better than the landowner themselves. This recognition is identified through the reframing process discussed earlier. Landowners perceive that if a forester is willing to navigate away from expert identity, then they may be willing to value landowner knowledge from a shared, personal frame.

Expert knowledge is imperative for the sustained health of forests – but foresters cannot know every detail of the landscape with immediacy. Landowners’ experiential knowledge serves to complete the puzzle of an ecological situation, and foresters can allow it to surface by engaging with and listening to them. In doing this, foresters will create that trusting, strong, and sustainable connection. This connection itself will contribute to landowners believing and continuing to engage with foresters, especially when they must do the hard work of navigating conflicts in thinking, goals, logistics, and ethics.

We recognize that stepping outside of identity rooted in tenure of successful expert practice, even for a moment, is confronting; but this work suggests it is vital to the continuation of that tenure as landowners and landscapes change. Continual self-reflection on personal and professional identity – and how these two are intrinsically linked – will be key for consultants who seek to engage landowners relationally. This implies the need to consider a shift from the traditional expert practitioner model, to a more reflexive, democratic one; wherein independent and collective reflection, alongside willingness to allow a landowner’s knowledge to expertly complement a forester’s in sound forest management, becomes foundational to an individual consulting forester’s practice. Both experience and expertise are sources of knowledge necessary to, and go hand-in-hand in, the care of forests. At times, foresters are experts and educators, and at times they are partners and students. The ability to move in and out of these roles is imperative to collaborative, sustainable forest management; it is the forester’s job to ensure that they do not check their empathy or know-how at the door.

## Conclusion

This research sought to understand the nature and impact of landowner–advisor interactions through landowners’ experiential stories. Results revealed that relationships and learning are integral tools for successful forest management. Knowing that failed landowner-forester connections have the potential to impact that management, findings reported in this research investigate the creation or negation of their relationships. Results suggest that the identity and perceptions foresters and landowners have of themselves and each other within an interaction dictate the likelihood of relationship formation. Further, when the two engage in interpersonal conversation beyond technicalities of management, they find sources of similarity that catalyze a reframing process and the creation of an effective, sustainable relationship. Without the discovery of similarity and trust, the foresters can remain within the frame of NLO skepticism, leading to a lack of engagement with professionals and subsequent potential for the forests to suffer. Implied within this dynamic is opportunity for consulting foresters to reflect on their perceptions of expert vs. personal identity and evaluate how to create personal connection with landowners within their practice. Taking that opportunity matters because, after all, being a forester *is* about more than being the expert – it’s about being a person, partner, and professional humbly, empathetically, and democratically carrying their valuable expertise.

## Footnotes

1 One forester type in a larger community of foresters in the state. They are business professionals who provide guidance and forest management services (e.g., write management plans, conduct timber harvest, implement invasive species treatment) to private forest landowners.

## References

[CR1] Andrejczyk K, Butler BJ, Dickinson BJ, Hews JH, Markowski-Lindsay M, Kittredge DB, Kilgore MA, Snyder JA, Catanzaro P (2016). Family forest owners’ perceptions of landowner assistance programs in the USA: a qualitative exploration of program impacts on behavior. Small-scale For.

[CR2] Brown TL, Lassoie JP (1998). Entry-level competency and skill requirements of foresters: what do employers want?. J For.

[CR3] Bullard SH, Moulton RJ(1988) The economics of public assistance for private nonindustrial timber sales in Mississippi.Faculty Publications.122. https://scholarworks.sfasu.edu/forestry/122

[CR4] Butler BJ, Hewes JH, Dickinson BJ, Andrejczyk K, Butler SM, Markowski-Lindsay M (2016). USDA Forest Service National Woodland Owner Survey: National, regional, and state statistics for family forest and woodland ownerships with 10 + acres, 2011–2013.

[CR5] Chhetri SG, Gordon JS, Munn IA, Henderson JE (2018). Factors influencing the use of consulting foresters by non-industrial private forest owners in Mississippi. Forestry Chron.

[CR6] Dzur AW (2018). Rebuilding public institutions together: professionals and citizens in a participatory democracy.

[CR7] Egan AF (1999). Reducing forest road erosion: do foresters and logging contracts matter?. J For.

[CR8] Egan A, Jones S (1993). Do landowner practices reflect beliefs? Implications of an extension-research partnership. J For.

[CR9] Elliott M, Gray B, Lewicki R, Lewicki R, Gray B, Elliot M (2003). Lessons learned about the Framing and Reframing of Intractable Environmental conflicts. Making sense of intractable environmental conflicts: concepts and cases.

[CR10] Erickson DL, Ryan RL, De Young R (2002). Woodlots in the rural landscape: landowner motivations and management attitudes in a Michigan (USA) case study. Landsc Urban Plan.

[CR11] Fischer AP, Klooster A, Cirhigri L (2019). Cross-boundary cooperation for landscape management: collective action and social exchange among individual private forest landowners. Landsc Urban Plan.

[CR12] Fischer F (2000). Citizens, experts, and the Environment: the politics of local knowledge.

[CR13] Flyvbjerg B, Sampson T (2001). Making Social Science Matter: why Social Inquiry fails and how it can succeed again.

[CR14] Gootee RS, Blatner KA, Baumgartner DM, Carroll MS, Weber WP (2010). Choosing what to believe about forests: differences between professional and non-professional evaluative criteria. Small-scale For.

[CR15] Gray B, Lewicki R, Gray B, Elliot M (2003). Framing of environmental disputes. Making sense of intractable environmental conflicts: concepts and cases.

[CR16] Hubbard WG, Abt RC (1989). The effect of timber sale assistance on returns to landowners. Res Mgmt & Optimization.

[CR17] Jamison A, Muth AB (2022) Forest landowners and advisor relationships: Creating collaborative connections to care well for forests, Society and Natural Resources. 10.1080/08941920.2022.2080311

[CR18] Kittredge D, Rickenbach M, Knoot T, Snellings E, Erazo A (2013). It’s the network: how personal connections shape decisions about private forest use. North J Appl For.

[CR19] Knoot TG, Schulte LA, Grudens-Schuck N, Rickenbach M (2009). The changing social landscape in the Midwest: a boon for forestry and bust for oak?. J For.

[CR20] Lewicki RJ, Gray B, Elliott M (2003). Making sense of intractable environmental conflicts: concepts and cases.

[CR21] McBride MF, Duveneck MJ, Lambert KF, Theoharides KA, Thompson JR (2019) Perspectives of resource management professionals on the future of New England’s landscape: Challenges, barriers, and opportunities. Landscape and Urban Planning 188 (2019): 30–42. 10.1016/j.landurbplan.2018.10.019

[CR22] Metcalf A, Finley J, Luloff AE, Muth AB (2012). Pennsylvania’s private forests: 2010 private forest landowner survey summary.

[CR23] Meyer J (2020). Are we producing society-ready foresters? A quantitative content analysis of graduate-level forestry curriculum. The Pegasus Review: UCF Undergraduate Research Journal.

[CR24] Palmer P (2011). Healing the heart of democracy: the courage to create Politics Worthy of the Human Spirit.

[CR25] Polkinghorne DE, Valle RS, Halling S (1989). Phenomenological Research Methods. Existential-phenomenological perspectives in psychology: exploring the Breadth of Human Experience.

[CR26] Pollio HR, Henley T, Thompson C (1997). The phenomenology of everyday life.

[CR27] Putnam L, Wondolleck J, Lewicki R, Gray B, Elliot M (2003). Intractability: definitions, dimension, and distinctions. Making sense of intractable environmental conflicts: concepts and cases.

[CR28] Redelsheimer C, Boldenow R, Marshall P (2015). Adding value to the profession: the role of accreditation. J For.

[CR29] Sample VA, Ringgold PC, Block NE, Giltmier JW (1999). Forestry education: adapting to the changing demands on professionals. J For.

[CR30] Sample VA, Bixler RP, McDonough MH, Bullard SH, Snieckus MM (2015). The promise and performance of forestry education in the United States: results of a survey of forestry employers, graduates, and educators. J For.

[CR31] Schön D, Rein M (1994). Frame reflection: toward the resolution of intractable policy controversies.

[CR32] Thomas SP, Pollio HR (2002). Listening to patients: a phenomenological approach to nursing research and practice.

[CR33] USDA Forest Service (2020) Forests of Pennsylvania, 2019. Resource Update FS-251. Madison, WI: U.S. Department of Agriculture, Forest Service. p. 2. 10.2737/FS-RU-251

[CR34] VanBrakle JD, Germain RH, Munsell JF, Stehman SV (2013). Do forest management plans increase best management practices implementation on family forests? A formative evaluation in the New York city watershed. J For.

[CR35] Wilson DC, Kilgore MA, Snyder S (2022). Planning and professional assistance as factors influencing private forest landowner best management practice implementation. J For.

